# Determining relative importance of variables in developing and validating predictive models

**DOI:** 10.1186/1471-2288-9-64

**Published:** 2009-09-14

**Authors:** Joseph Beyene, Eshetu G Atenafu, Jemila S Hamid, Teresa To, Lillian Sung

**Affiliations:** 1 Child Heath Evaluative Sciences, The Hospital for Sick Children, Toronto, Canada; 2 Division of Hematology/Oncology, The Hospital for Sick Children, Toronto, Canada; 3 Dalla Lana School of Public Health, University of Toronto, Toronto, Canada; 4 Department of Health Policy, Management and Evaluation, University of Toronto, Toronto, Canada

## Abstract

**Background:**

Multiple regression models are used in a wide range of scientific disciplines and automated model selection procedures are frequently used to identify independent predictors. However, determination of relative importance of potential predictors and validating the fitted models for their stability, predictive accuracy and generalizability are often overlooked or not done thoroughly.

**Methods:**

Using a case study aimed at predicting children with acute lymphoblastic leukemia (ALL) who are at low risk of Tumor Lysis Syndrome (TLS), we propose and compare two strategies, bootstrapping and random split of data, for ordering potential predictors according to their relative importance with respect to model stability and generalizability. We also propose an approach based on relative increase in percentage of explained variation and area under the Receiver Operating Characteristic (ROC) curve for developing models where variables from our ordered list enter the model according to their importance. An additional data set aimed at identifying predictors of prostate cancer penetration is also used for illustrative purposes.

**Results:**

Age is chosen to be the most important predictor of TLS. It is selected 100% of the time using the bootstrapping approach. Using the random split method, it is selected 99% of the time in the training data and is significant (at 5% level) 98% of the time in the validation data set. This indicates that age is a stable predictor of TLS with good generalizability. The second most important variable is white blood cell count (WBC). Our methods also identified an important predictor of TLS that was otherwise omitted if relying on any of the automated model selection procedures alone. A group at low risk of TLS consists of children younger than 10 years of age, without T-cell immunophenotype, whose baseline WBC is < 20 × 10^9^/L and palpable spleen is < 2 cm. For the prostate cancer data set, the Gleason score and digital rectal exam are identified to be the most important indicators of whether tumor has penetrated the prostate capsule.

**Conclusion:**

Our model selection procedures based on bootstrap re-sampling and repeated random split techniques can be used to assess the strength of evidence that a variable is truly an independent and reproducible predictor. Our methods, therefore, can be used for developing stable and reproducible models with good performances. Moreover, our methods can serve as a good tool for validating a predictive model. Previous biological and clinical studies support the findings based on our selection and validation strategies. However, extensive simulations may be required to assess the performance of our methods under different scenarios as well as check their sensitivity to a random fluctuation in the data.

## Background

Regression models have long been used in medicine, clinical epidemiology, health services research, pharmaceutical research, social sciences and business studies. Their application has greatly increased in the past few decades [1-3]. In particular, regression models such as logistic and Cox models have become standard statistical methods in medicine and epidemiology [4]. Regression models can also be used to combine data from different sources. In cancer research, for example, predictive models based on wide range of data such as clinical, gene expression and single nucleotide polymorphism (SNP) are frequently developed to assess risk factors with the ultimate aim of developing better prevention, diagnostic and treatment strategies [5-8]. In recent years, clinical data have been integrated with genomic and other types of data for developing more accurate predictive models [9-11]. For instance, clinico-genomic models were used in breast cancer outcomes prediction where genomic data combined with clinical and demographic characteristics were used for improving predictive accuracy [11].

Despite the fact that predictive models are commonly used in wide range of scientific disciplines, they are frequently presented inadequately [3,12,13]. A recently published systematic review discusses the gravity of this issue [12]. The authors investigated 2,234 papers published in 104 journals in areas of obstetrics and gynecology. A considerable percentage (34.2%) of the studies used logistic regression. Among those that utilized logistic regression, most (96%) reported significance (in the form of p-values), however, only 3.6% reported goodness of fit. None of them considered model validation of any sort. The authors, however, did not distinguish between etiologic and predictive models. The empirical results, therefore, might well include both types of models. Concerns regarding such inadequate presentation of predictive models in other areas of medicine such as cancer research have also been reported [3,13-15].

Validity of predictive models as well as their application in the general population highly depends on their goodness of fit [3,4,16,17]. Assessing goodness of fit and model validation are, therefore, essential in any model fitting task in general and in constructing predictive models in particular.

There are different methods for developing predictive models and it is very challenging to maintain balance between including too many variables (and risk loss of precision) and omitting important variables (and risk biased prediction) [18]. Automated variable selection methods are frequently used in regression analysis for selecting important predictors, however, these approaches may result in spurious noise variables being mistakenly identified as independent predictors of the outcome [19]. Moreover, predictive models developed using such automated algorithms tend to be non-reproducible and hence are not reliable in practice. Use of good strategies for model selection along with adequate performance and goodness of fit measures are, therefore, needed in developing accurate prediction.

The ultimate goal of developing a predictive model is to use the model for predicting future outcomes in a much larger, broader and heterogeneous population. It is important to note that accurate models that perform extremely well may perform poorly outside the data set they are built on. In fact, the performance of a predictive model is overestimated if determined on data used to construct the model and may not be reliable for the larger population [17]. For more details regarding model selection and the tradeoff between model over-fitting and under-fitting can be found in [20,21].

In this paper, we propose two different approaches for ordering potential predictors according to their relative importance with respect to model stability and generalizability. We use the concept of explained variation and area under the Receiver Operating Characteristic (ROC) curve for developing models and in determining how many predictors to include in final model. We illustrate the approaches using a study conducted by Truong and colleagues [5]that aimed at developing a prediction rule to identify patients with Acute Lymphoblastic Leukemia (ALL) at lower risk of Tumor Ly sis Syndrome (TLS). A publicly available data set is also used for additional illustration.

## Methods

### Motivating case studies

To illustrate model selection and validation methods presented in this paper, data and statistical results from previous two studies are considered. Here we give brief descriptions of the original studies. For more details about the data and the statistical analyses performed, we refer the reader to the original papers [5,22]. We focus on the first case study and illustrate our methods using this data set. However, data from a prostate cancer study is also considered for additional and supplementary illustration.

#### Case Study 1 - Prediction of children at low risk for Tumor Lysis Syndrome (TLS)

With the long-term aim of a risk-stratified approach to the prevention of Tumor Lysis Syndrome (TLS), the study was originally performed to describe the prevalence and predictors of TLS in childhood Acute Lymphoblastic Leukemia (ALL) and develop a sensitive prediction rule to identify patients at low risk of TLS [5].

In this paper, we consider the primary outcome, i.e., presence or absence of TLS. In total, 328 patients (of which 74 are TLS cases) were included in the study. Potential predictors included in the model consist of 1) Laboratory features: white blood cell count (WBC ≥ 20 × 10^9^/L) and presence of T-cell immunophenotype 2) Clinical indicators: presence of mediastinal mass, hepatomegaly(palpable liver ≥ 3 cm) and splenomegaly (palpable spleen ≥ 2 cm), and 3) Demographic characteristics: age (≥ 10 years) and sex.

#### Case Study 2 - Prostate Cancer Study

The main objective of the original study was to determine whether variables measured at a baseline exam can be used to predict whether the tumor has penetrated the prostate capsule. A total of 380 subjects are considered of which 153 had a cancer that penetrated the prostate capsule. Variables included in the study are age, race, results of digital rectal exam(DPROS), detection of capsular involvement in rectal exam(DCAPS), prostate specific antigen (PSA), tumor volume from ultrasound (VOLUME) and total Gleason score. We refer the reader to [22]for more details about this data set.

### Proposed methods and analytical strategies

We propose two approaches for ordering variables according to their relative importance with respect to model stability and generalizability. A predictive model is then constructed using explained variation and area under the Receiver Operating Characteristic (ROC) curve where variables from our ordered list enter the model according to their relative importance. Increase in explained variation as well as the area under the ROC curve is used to determine the number of variables included in the final model.

#### Bootstrapping for model selection

Bootstrapping is a modern, computer-intensive re-sampling approach which allows simulation of test data sets that mimic the initial original data set and the technique has been used successfully in a wide range of applications [23]. An excellent comprehensive tutorial paper that focuses on the application of the bootstrap method for constructing confidence intervals is provided in [24]. Bootstrap re-sampling along with an automated selection procedure have been used to develop parsimonious models [25,26]. It has been demonstrated that bootstrap methods can be used to assess the strength of evidence that an identified variable truly is an important predictor. Sauerbrei and Schumacher used bootstrap sampling to assess the distribution of an indicator variable denoting the inclusion of a specific predictor for identifying strong and weak factors for predicting survival [26]. Bootstrapping has also been used to show that automated model selection procedures are likely to identify noise variables as important predictors and hence produce unstable models in a logistic regression setup [27].

Here we propose the use of bootstrapping for ordering potential predictors according to their importance. We took one thousand bootstrap samples from the original data. On each bootstrap sample, we applied three most commonly used automated model selection procedures (forward, backward and stepwise) where all candidate variables are included at the initial stage of the analysis. Candidate variables are, then, ordered according to their importance, where the variable chosen most frequently is ranked first.

After the initial ordering of the candidate predictors using bootstrapping, our final predictive model is constructed using a stepwise procedure where the top ranked variable from our ordered list enters the model first. Variables are then sequentially added to the model according to their relative importance. At each stage, we calculate the improvement in the predictive accuracy of the model, measured by the percentage of explained variation and area under the ROC curve.

#### Random Split of Data

The bootstrap approach described above addresses the issue of variable stability. However, model reproducibility is also another important criterion since models are often developed with the aim of predicting future outcomes. We considered variables to be reproducible or generalizable if their predictive power can be generalized to new data set. That is, the model consisting of these variables not only have a good predictive power for the data set the model is built on, but also its performance (with respect to predictive power) can be generalized in predicting future outcomes. Here we propose an approach for model selection that not only considers model stability but also generalizability and reproducibility. We randomly split the original data evenly into training and validation data sets. This step is repeated one thousand times. We use the training data sets to build the models and validate the selected models using the validation data sets. All candidate variables are considered and potential predictors are selected using forward, backward and stepwise selection procedures using the training data sets. Moreover, for each random split of data, we checked if the selected predictors are significant in the validation data set. The variables are then ordered according to their relative importance, where the importance of a variable is measured by the proportion (s) it is selected as a predictor and as well as the proportion (*v*) at which it is significant in the validation data set. We propose the product *sv *as a measure of relative importance incorporating stability and generalizability. This product is equivalent to the proportion at which a variable is both selected in the final model and is significant using the validation data set.

That is, if we define two events such that A = variable is selected using an automated model selection procedure based on the training data set, B = variable is significant using the validation data set. Then, A & B = variable is selected using the training data set and it is significant in the validation data set. What we are interested in is the probability of A & B. This probability, which incorporates stability and generalizability measures, can be estimated as the proportion at which a given variable is selected and is significant simultaneously. It is possible to show that this proportion is equivalent to s*v*.

#### Automated model selection procedures

Automated model selection procedures: backward elimination, forward selection and stepwise selection, are the three most commonly used variable selection procedures. In backward elimination, variables are eliminated from the full model based on a certain criteria mostly based on a decrease in R^2^or deviance. In forward selection, however, we start with an empty model and variables are added sequentially where, at each step, a variable that brings the largest increase in R^2^or deviance will be added in the model. Stepwise procedure is a variation of forward selection where variables are allowed to be eliminated from the model. The stopping rule, which is usually pre-specified, for all the three automated model selection procedures is based on significance at a specified level. For comprehensive overviews of automated model selection procedures, we refer the reader to [28,29]. For variable selection from a Bayesian perspective we refer the reader to [30].

#### Percentage of explained variation

We use two measures of explained variation appropriate for binary outcome - direct and indirect [16]. The direct measure is based on residual from the fit and the indirect index is related to standard measure of information. Suppose the estimates from a logistic model without a covariate (i.e., unconditional) are denoted by  and let the estimates from a model with covariate x_i _(i.e., conditional) be . Define  and . The explained variation based on the direct estimates is given by . Similarly, let  and . The explained variation based on the indirect estimates is calculated as .

#### Five - fold cross validation

We performed 5-fold cross validation based on the original TLS data to check the performance of our approaches. That is, we randomly divided the data into five parts, estimate model parameters based on 4 of the 5 data sets and use the remaining one fold for validation. The randomization is repeated 5 times and prediction error is averaged over the five cross validations and the number of randomizations. By doing repeated cross-validation, it is possible to reduce variability and provide more stable estimates.

## Results

### Case study 1

Table 1 shows results from the bootstrapping approach in which covariates are ordered by the proportion at which they were selected in the final model following three commonly used selection procedures: forward, backward and stepwise. The p-values presented in the table are averaged over 100 bootstrap samples. It can be seen that the order of importance, where the variable chosen most frequently is considered as the most important predictor, is the same for all the three selection procedures. Age is chosen almost 100% of the time indicating that it is the most important predictor. Hepatomegaly is chosen the least in all of the three procedures, chosen about 50% of the time using the forward method and 30% of the time using backward and stepwise selection techniques. Moreover, the results show that all of the seven variables considered are significant (at 5% level)in the backward and stepwise selection procedures, once chosen as predictor. However, mediastinal mass and hepatomegaly are not statistically significant using the forward selection procedure. Overall, our analysis shows that all the three automated procedures, followed by bootstrapping, produce similar results.

**Table 1:** Proportion (s) at which each of the variables entered the final model and average p-value for three automated model selection procedures based on one thousand bootstrap samples

Variables	Forward selection		Backward selection		Stepwise selection	
	*s*	p-value	*s*	p-value	*s*	p-value
Age	1.000	0.0003	0.999	0.0002	0.998	0.0003
Baseline WBC	0.991	0.0067	0.957	0.0028	0.951	0.0027
Splenomegay	0.767	0.0424	0.600	0.0071	0.610	0.0071
T-cell immunophenotype	0.748	0.0408	0.593	0.0043	0.588	0.0042
Sex	0.719	0.0493	0.465	0.0141	0.455	0.0137
Mediastinal mass	0.648	0.0576	0.450	0.0056	0.451	0.0055
Hepatomegaly	0.493	0.0621	0.307	0.0111	0.292	0.0108

After the initial ordering of the candidate predictors using bootstrapping, our final predictive model for identifying patients at low risk for TLS is constructed using a stepwise procedure where the top ranked variable enters the model first. We calculate the improvement in the predictive accuracy of the model, measured by the percentage of explained variation and area under the ROC curve, at each stage to see if the next variable needs to be included in the model. Table 2 shows two estimates of percentage of explained variation(direct and indirect) and area under the ROC curve obtained by including the variables in the model according to the order of importance given in Table 1.

**Table 2:** Cumulative percentage of explained variation and area under ROC obtained by including the variables in the model according to the order of importance as given in Table 1.

Variables	Percentage EV_direct_	Percentage EV_indirect_	Percentage area under ROC curve
Age	8.65	8.65	64.3
Baseline WBC	18.70	19.04	75.7
Splenomegay	21.35	21.80	79.4
T-cell immunophenotype	27.18	27.05	81.3
Sex	29.54	29.42	82.7
Mediastinal mass	30.50	30.38	82.8
Hepatomegaly	30.73	30.76	83.2

A model with the first four variables explained 27.18% with area under ROC curve 81.3%. The increases in percentage variation as well as area under ROC curve are negligible after the first four variables have been included in the model. A final model with a good predictive accuracy (area under ROC = 81.3%) can be constructed using the first 4 variables. Group of children at low risk of TLS can, therefore, be defined as absence of age, baseline WBC, splenomegaly and T-cell immunophenotype. That is, children younger than 10 with absence of T-cell immunophenotype, whose baseline WBC < 20 × 10^9^/L and a palpable spleen < 2 cm, are at low risk for TLS. It is important to note that this model is different from the one constructed in the original study where the predictors in the final model are age, baseline WBC, splenomegaly and mediastinal mass. The last variable is the second last variable in our ordering.

As can be seen from Table 2, the percentage of explained variation increases sharply in the beginning. For instance, a model with only age explained almost 9% of the variation with area under ROC 64.3, whereas age and baseline WBC combined explained 19% of the variation with area under ROC 75.7.

The results from the random split approach where variables are ordered according to *sv*, a measure incorporating stability and generalizability, are presented in Table 3. Out of 1000 random splits of data, age was selected 994, 974 and 971 times using forward, backward and stepwise selecting procedures. Out of the 994 inclusions based on forward selection using the training data set, age was significant in the validation data set 979 times (*s *= 979/994 = 0.985).

**Table 3:** The proportion at which a given variable is selected in the training set and is significant in the validation set after randomly splitting the data 1000 times

Variables	Forward selection	Backward selection	Stepwise selection
	*sv*	*sv*	*sv*
Age	0.979	0.956	0.954
Baseline WBC	0.873	0.803	0.789
T-cell immunophenotype	0.394	0.464	0.464
Mediastinal mass	0.248	0.270	0.273
Splenomegay	0.192	0.118	0.136
Hepatomegaly	0.016	0.000	0.000
Sex	0.000	0.000	0.000

The same ordering is obtained using all of the three model selection procedures where age and baseline WBC are selected as the first two important predictors (Table 3). Recall that this was also the case using the bootstrapping approach. However, it is important to note that different orderings are obtained using the two procedures in general. This is to be expected since the random split approach incorporates information regarding the generalizability of the model (via the validation stage) in addition to model stability provided by the bootstrapping. For instance, splenomegaly is the 3rd important variable using bootstrapping, however, it is the 5^th ^selected variable in the training set using the random split strategy. This is because, even if it was selected 61% of the time, it was significant only 31.1% of the time in the validation set (and hence lacks generalizability). Overall, the proportion it is selected in the training data set and significant in the validation data set simultaneously is only 0.192. Now that we have ordered our variables according to their importance with respect to model stability and generalizability, the next question is to determine which of the variables to include in the final model. It is already obvious to see, as indicated in Table 3, that hepatomegaly and sex are not significantly important variables in predicting children at low risk for TLS. However, we use the concept of explained variation and area under ROC as before to select the final model. The results from the analysis are presented in Table 4.

**Table 4:** Percentage of explained variation and area under ROC obtained by including the variables in the model according to the order of importance given in Table 3.

Variables	Percentage EV_direct_	Percentage EV_indirect_	Percentage area under ROC curve
Age	8.65	8.65	64.3
Baseline WBC	18.70	19.04	75.7
T-cell immunophenotype	25.01	24.96	79.1
Mediastinal mass	26.25	26.14	79.8
Splenomegay	28.22	28.05	81.5
Hepatomegaly	28.51	28.38	81.8
Sex	30.73	30.76	83.2

The results presented in Table 4 indicate that about 25% of the variation is explained by the first three variables and the area under the ROC curve for a predictive model consisting of only these three variables is 79.1. Table 4 show s that the increase in the percentage of variation as well as the area under the ROC curve is very small after including the first 3 variables.

The model with the first 5 variables explains about 28% of the variation and the area under the ROC curve is 81.5. However, the contribution of mediastinal mass towards the total variation as well as area ROC is very small once T-cell immunophenotype is included in the model. This might be an indication that these two variables are highly correlated and hence some of the contribution of mediastinal mass is already accounted for by T-cell immunophenotype. We used a chi-square test to investigate the relationship between these two variables and the results indeed show a very strong association (p-value < 0.0001). As a result, including both of the variables in the final model is not only unnecessary but also introduces multicolinearity to the model. From Tables 1 and 4, we can see that T-cell immunophenotype is more important than mediastinal mass according to importance measures related to model stability and generalizability. We, therefore, keep age, baseline WBC, T-cell immunophenotype and splenomegaly in the final model. In practice, however, further biological/clinical investigation might be required to assess the biological and/or clinical importance of the variables involved before we decide to exclude variables from the final model. According to our statistically derived and validated model, a group at low risk of developing TLS consists of children younger than 10 years, without of T-cell immunophenotype, whose baseline WBC < 20 × 10^9^/L and palpable spleen < 2 cm. Note that this low risk group is similar with the group selected using the bootstrap approach.

It is also important to note that, T-cell immunophenotype, which is selected as the third most important variable using the random split and the fourth important variable in the bootstrapping was excluded from the model proposed in the original paper [5].

We used 5-fold cross validation to assess the performance of the models elected based on the bootstrapping and random split approaches. Recall that both approaches produced the same model - a predictive model consisting of age, baseline WBC, T-cell immunophenotype and splenomegaly. We also consider the model proposed in the original paper for comparison purposes. Their model is based on age, baseline WBC, Mediastinal mass and Splenomegaly [5].

The results from our analysis show that the model selected using our approach has a slightly less prediction error (0.17) than the model proposed in the original paper (0.19). The corresponding ROC values for the two models are 81.3% and 81.1%, respectively. This indicates that the model proposed in the original paper performs well in general and has good generalizability. However, an important variable was omitted from the model as discussed earlier. Moreover, the original model consisting of age, baseline WBC, mediastinal mass and splenomegaly, was not validated although some goodness of fit properties were reported. The approaches proposed in this paper and the statistical results from the analysis can, therefore, be used as a confirmation for the performance of the proposed model and its appropriateness for practical use.

### Case study 2

**Figure 1:**
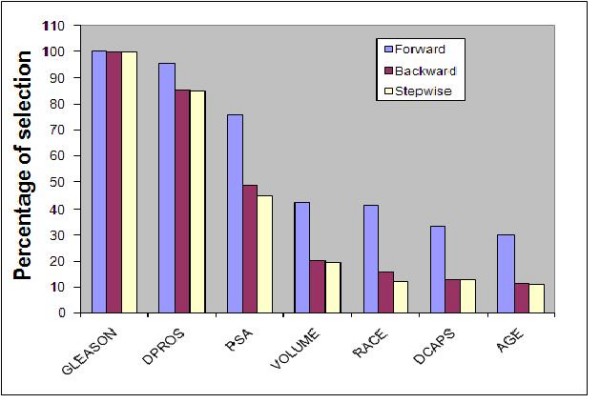
**Relative importance of variables for the prostate cancer data, measured using the percentage of selection out of 1000 bootstrap samples**.

**Figure 2:**
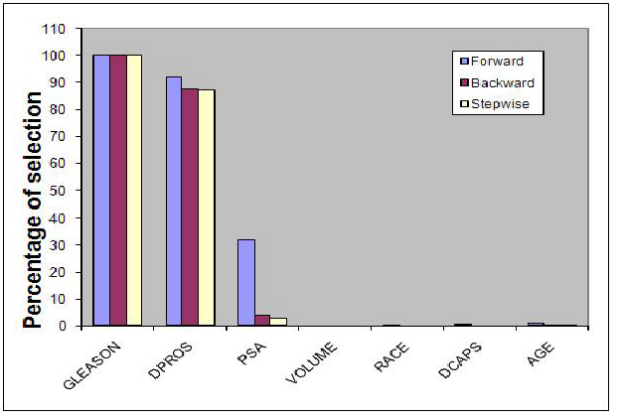
**Relative importance of predictors for the prostate cancer data using the random split approach**.

As a second case study we considered a prostate cancer data set briefly described in the previous section. Bar graphs showing relative importance of variables using our two approaches are presented in Figures 1 and 2, respectively. The bootstrap method identified two variables (Gleason score and results from digital rectal exam(DPROS)) as important predictors of prostate cancer penetration where they are selected 99.6% and 84.8% of the time, respectively, out of 1000 samples based on stepwise selection. These variables are considered by clinicians as two of the most influential factors used to determine treatment for prostate cancer and have also been previously identified as predictors of death in prostate cancer [31,32]. Prostate specific antigen (PSA) value is also identified as an important predictor based on forward selection where it is selected 75.9% of the time. However, this variable is selected less than 50% of the time using backward and stepwise methods. This is not surprising since forward selection is often optimistic since the selection process, unlike the stepwise and backward selection methods, does not involve an elimination step even if the variable already included does not contribute much in the presence of other variables. Our results from the analysis of explained variation and area under the ROC curve are also in agreement with this conclusion. That is, using 5 fold cross validation, the increase in the explained variation as well as the area under the ROC curve when PSA is added to the model is small (ROC from 80.9% to 82.3%).

Since the data is relatively larger than case study 1 and also has a larger event rate, we divided the data set in to training and validation where we set aside 1/3^rd^of the data for validation purposes. For the training set, a model consisting of the first two important variables, Gleason score and digital rectal exam, gave area under the ROC = 84.2%. This value increased to just 85.3%when PSA is added to the model. When testing using the validation data(the 1/3 ^rd ^data set aside in the beginning), the area under the ROC increased from 74.2% to 75.6% indicating that although PSA seems to be an independent predictor for this particular data set, it may not be a good predictor for future data and hence non reproducible. This finding is supported by previous studies that have shown that, although PSA is negatively associated with cancer penetration, it was not able to predict pathological features in clinically localized prostate cancer in the overall population [33,34].

## Discussion

Predictive models are used frequently in medical research and automated model selection procedures are traditionally used for identifying predictors. However, assessing model fit and validation of the selected models are rarely performed or not done thoroughly, if done at all. Moreover, models selected using automated procedures often include noise variables that have little or no relationship with the outcome variable. Similarly, important variables might be omitted.

In this paper we proposed two approaches for ordering candidate variables according to measures incorporating model stability and generalizability. In our first approach, we used bootstrapping and defined variable importance as the proportion a given variable is selected and hence leads to models that are more stable than models selected using only automated procedures. This method is similar to that of Austin and Tu [26,27]. What distinguishes our approach from theirs is that we use d measures of importance such as relative increase in percentage of explained variation and area under the ROC curve in determining the number of variables included in the final model. In the second approach, we performed one thousand random split of data into training and testing data sets. Variables are then ordered according to the frequency they were selected using the training data set at the same time statistically significant in the validation data set. Our second approach, thus, leads to a ranking that not only incorporated model stability but also takes generalizability and reproducibility into account. We would like to note that random split, like cross validation, involves random sampling. The underlying population is, therefore, the same. When available, a more rigorous test based on a completely different sample from another population can be used to assess model generalizability. How to do the random split is also an important issue that has been addressed by many in classification/prediction literature. The amount of data to set aside often depends on the sample size and also predictive ability of the variables included in the analyses. For a small sample size, for instance, 50% split as done in our manuscript might lead to poor predictive model (classifier) since the model is built on insufficient data set. Under such circumstances, setting 1/3rd of data aside for testing might give better results. One could also use k-fold cross validation (where k > 2) when the sample size is small.

We used the concept of explained variation and area under ROC where the most important variable enters the model first and variables are added until the improvement in explained variation as well as area under the curve is negligible. This is particularly important when the number of candidate variables is very large and one has to select the first top most important variables. The issue of how small the improvement has to be, in order to decide the number of variables entering the final model, is a very challenging task. In this paper, we used an approximate cutoff point using plots similar to scree plot in principal component analysis which allows us to visually assess the point where the improvement becomes negligible. However, it might be interesting to develop a test statistic based on the percentage increase in explained variation and/or area under the ROC curve. One can, thus, test if the improvement is statistically significant or not. This is a topic we would like to investigate in future research.

It is important to note that, the original childhood leukemia study presented in this paper is well designed where candidate predictors were selected after a thorough investigation of their clinical relationship with the outcome variable. Consequently, not much difference was observed between the performance of the original model and the model constructed using our approaches. However, the results in this paper indicate that even in such well designed study an important variable could be omitted if relying on automated model selection procedures alone. This indicates that researchers should be more cautious when using automated procedures and they are encouraged to use re-sampling techniques along with these procedures.

Application of our approaches to the prostate cancer data set identified two variables as important predictors of whether tumor has penetrated the prostate capsule. These variables have been previously identified as powerful indicators of tumor penetration and prostate cancer death supporting our findings. Moreover, prostate specific antigen (PSA), which was ranked as the third important variable based on the bootstrap approach, was not identified as an important variable in using our random split approach. This indicates that PSA, although associated with prostate cancer, is not a reproducible predictor of tumor penetration and hence not helpful for predicting for patients outside the study population. This finding is also supported by previous biological and clinical studies.

## Conclusion

In conclusion, automated model selection procedures may result in the inclusion of noise variables and unstable models. Our proposed methods and analytical strategies can be used to assess variable stability and reproducibility and hence may lead to more robust models with favorable predictive performance. However, extensive simulation is needed to study the operating characteristic of our approaches in a more general setting. We plan to pursue this in future research.

## Competing interests

The authors declare that they have no competing interests.

## Authors' contributions

JB contributed to the conception and design of the study, interpretation of data and drafting of the manuscript. EGA contributed to the conception and design of the study, analysis and interpretation of data. JSH contributed to the conception and design of the study, interpretation of data and drafted the manuscript. TT helped with critical revision of the manuscript for important intellectual content. LS contributed to the conception of the study, acquisition of data and helped with critical revision of the manuscript for important intellectual content. All authors read and approved the final manuscript.

## Pre-publication history

The pre-publication history for this paper can be accessed here:

http://www.biomedcentral.com/1471-2288/9/64/prepub
